# Transitions experienced by people living with limitations resulting from leprosy: a research-care study

**DOI:** 10.1590/0034-7167-2023-0229

**Published:** 2024-11-22

**Authors:** Rayla Maria Pontes Guimarães Costa, Marcia Astrês Fernandes, Ivete Palmira Sanson Zagonel

**Affiliations:** I Universidade Estadual do Piauí. Parnaíba, Piauí, Brazil; II Universidade Federal do Piauí. Teresina, Piauí, Brazil; III Universidade Federal do Paraná. Curitiba, Paraná, Brazil

**Keywords:** Leprosy, Disabled Persons, Adaptation, Psychological, Transitional Care, Quality of Life, Lepra, Personas con Discapacidad, Adaptación Psicológica, Cuidado de Transición, Calidad de Vida

## Abstract

**Objective:**

to understand the transitional processes that affect the adaptation of people who live with limitations resulting from leprosy.

**Methods:**

This is a qualitative study based on the precepts of Transition Theory, mediated by care-research, with 24 people with limitations resulting from leprosy in an ex-hospital colony in Piauí. Semi-structured interviews were carried out. The interviews were analyzed using Iramuteq software.

**Results:**

the researched-caregivers experienced the four types of transitions, including feelings of fear, worry, loneliness, hopelessness, guilt and a tendency to hide the diagnosis. Breakdowns and resignation were revealed, with spirituality, adaptation to the new life situation and acceptance as facilitating conditions for coping with the transitional process, with a consequent improvement in quality of life.

**Final considerations:**

the transitional processes had a positive significance, since they contributed to adaptation and the achievement of quality of life.

## RESUMEN

**Objetivo:**

comprender los procesos de transición que inciden en la adaptación de personas que conviven con limitaciones derivadas de la lepra.

**Métodos:**

es una investigación de enfoque cualitativo, basada en los preceptos de la Teoría de Transición y mediada por la investigación en cuidados. Se llevó a cabo mediante entrevistas semiestructuradas entre 24 personas con limitaciones derivadas de la lepra, en un antiguo hospital colonia de Piauí, Brasil y se analizó con el *software Iramuteq.*

**Resultados:**

los investigados cuidados experimentaron los cuatro tipos de transiciones, incluyendo sentimientos de miedo, preocupación, soledad, desesperanza, culpabilidad y tendencia a ocultar el diagnóstico. Salió a la luz la ruptura y la resignación, aunque también la espiritualidad, la adaptación a la nueva situación vital y la aceptación como condiciones facilitadoras para afrontar el proceso de transición, con la consiguiente mejora de la calidad de vida.

**Consideraciones Finales:**

los procesos de transición demostraron un significado positivo al contribuir con la adaptación a su realidad y así lograr una mejor calidad de vida.

## INTRODUCTION

Leprosy is an infectious and contagious disease caused by the bacillus Mycobacterium leprae, which attacks the skin, mucous membranes and peripheral nerves, and has the potential to cause neural lesions with irreversible damage. These neural lesions triggered by leprosy are responsible for disability, leading to stigma and discrimination. Leprosy is a major public health problem in Brazil, which currently ranks second in the world among countries registering new cases^(1)^.

In addition, leprosy is still actively transmitted in several countries, most notably India, Brazil and Indonesia, which are responsible for around 74.5% of the total cases recorded in 2021 (140,594). Each of these countries reported more than 10,000 cases^(2,3)^, which requires special attention, since the disease can evolve with limitations.

The limitations resulting from leprosy affect the patient’s daily life, as they impact on the performance of activities of daily living, requiring attention from health professionals even before treatment begins^(4)^. In fact, it is imperative to develop the professional practice of nursing with people who live with limitations resulting from leprosy, listening and providing professional care, considering subjectivities, understanding the transitions experienced in the face of changes that require coping and adapting to the new life situation.

Adapting to the new situation can happen late or suddenly, in conjunction with the changes that occur throughout the life cycle, caused by life events^(5)^, that can affect the quality of life of the person in transition.

Transition can be conceptualized as the passage from one phase, place, condition/state and/or subject to another, as a result of a change. Transition, as a process and a result, arises from the interaction between person and environment, and is aggregated in a context and/or situation, expressing a change in health status or in role relationships, expectations or abilities^(6)^, being related to other concepts such as adaptation, adjustment and self-care^(7)^, in which the responses to this process are manifested by health-related behaviors^(8)^.

Thus, it was possible to conjecture a gap in comprehensive care for people with limitations resulting from leprosy from the perspective of the transitions that occurred in the illness process, which compromised well-being and quality of life, greatly affecting the way these people live, which can cause vulnerability and marks on self-esteem, cognitive and neuropsychiatric aspects.

The study also has scientific and social relevance. Scientifically, it is innovative and contributes significantly to the advancement of science. Socially, it addresses the unfolding of an ancient disease which, to this day, continues to have a negative impact on those affected. It even has a direct impact on quality of life and can cause both physical and psychological damage. On the physical side, it interferes with carrying out daily and work tasks, while on the psychological side it affects social relationships in the environment in which people live, and can lead to exclusion, loneliness and even depression^(9)^.

In this way, we sought to focus on the transitions experienced by people with limitations resulting from leprosy throughout their lives. In this context, the object of study was defined as the transitions experienced by people living with limitations resulting from leprosy and the adaptation to changes, from the perspective of care research. It is assumed that the object will subsidize the construction of new knowledge and unravel doubts inherent in the transitions experienced. In addition, the proposed study aims to answer the following guiding question: what is the significance of the transitional processes that affect the adaptation of people who live with limitations resulting from leprosy through care-research?

## OBJECTIVE

To understand the transitional processes that affect the adaptation of people living with limitations resulting from leprosy.

## METHODS

### Ethical aspects

The study was conducted in accordance with national and international ethical guidelines and approved by the Research Ethics Committee of the Federal University of Piauí (UFPI). Ethical precepts were complied with in the execution and development of the research, the opinion of which is attached to this submission.

### Type of study and theoretical-methodological framework

A qualitative research approach anchored in the precepts of Transition Theory, a mid-range nursing theory based on the transitional processes experienced throughout the human life cycle^(8)^, mediated by the methodological aspects of research-care. Care-research can be developed in five stages: approaching the object of study; meeting the researcher (caregiver) with the researched being (care); establishing the connections between research, theory and care practice; distancing the researcher and researched beings and analyzing what has been grasped^(10)^. All the stages of research-care were used in this study.

It should be emphasized that for research-care it is essential that the nurse (being a researcher) ensures a relationship of care with the being researched/caregiver, where research and care are part of the same context, with this, the nurse, at one moment, listens and records and, at another, cares and does not record, cares and records, educates, manages, that is, research while caring-teaching-managing^(11)^. In addition, the recommendations for the dissemination of qualitative studies proposed by the Consolidated criteria for reporting qualitative research (COREQ) guide were followed^(12)^.

### Study scenario

The study was carried out in a former colony hospital, a state public hospital, a reference in the treatment of leprosy in the state of Piauí, located in the municipality of Parnaíba, Piauí.

### Data source

The study participants were 24 people aged 18 and over, who have limitations as a result of leprosy. The inclusion criteria were: having permanent sequelae with varying degrees of disability as a result of leprosy and being willing and interested in taking part in the study. Those who were absent from the study site during the data collection period were excluded from the study.

### Data collection and organization

Data collection took place between June and September 2021, using semi-structured face-to-face interviews. A script with the following topics was used to guide the interview: meaning of the diagnosis of leprosy, living with the limitations resulting from leprosy, alterations/changes observed in life as a result of the limitations of leprosy, evaluation of the quality of life after the diagnosis of the disease, problems experienced as a result of the limitations that affected the quality of life and strategies used to overcome the problems/changes, and also, data that included the sociodemographic profile (age, gender, marital status, schooling, occupation, income and religion).

The interview took place in individualized meetings held at the institution. At this meeting, the theme and aim of the research was explained, as well as the information collection technique that would be adopted. They were invited to take part in the research, and if they accepted, the interview was scheduled for a date and time that suited the researcher-caregiver, and they were asked to agree to take part in the study. The Free and Informed Consent Form (FICF) was obtained from all the researched-caregivers in writing. The interviews were carried out in a private place, as scheduled. At the end of the interview, the individualized practice of transitional care was carried out. The interviews were recorded on an MP4 device and transcribed in full.

This was also the moment when the care-research itself took place, in which there was interaction between the researcher and the researched, in which the researcher-caregiver sought to capture and unveil the meanings attributed by people living with limitations resulting from leprosy to the transitions experienced and adaptation from the perspective of achieving quality of life. Care was provided in a variety of ways: listening, looking, closeness, respect, inclusion, health guidelines and follow-up. At this point, emotions were expressed, such as sadness and joy, as they recalled aspects of their own lives, in other words, their experiences and reactions to certain situations.

Thus, the data was organized into two thematic axes and presented in five classes, with the respective needs learned during the research-care.

### Data analysis

The information from the interviews was recorded and transcribed in full. They were then analyzed using the Iramuteq software (Interface de R pourles Analyses Multidimensionnelles de Textes et de Questionnaires, version 0.7), a free and open source program created by Pierre Ratinaud that allows qualitative data to be explained graphically^(13)^.

Initially, the interviews were transcribed and organized into a single textual corpus. To do this, some words were grouped together in order to improve the analysis. Other words such as quality of life, problems experienced and changes were kept. When organizing the material for analysis, sociodemographic variables such as age, gender, marital status, education, occupation, income and religion were included in the command line and presented in a table.

The Descending Hierarchical Classification (DHC) was used for the analysis and the data was presented in a figure. Furthermore, in view of the robustness of the Descending Hierarchical Classification (DHC), authors indicate a minimum retention of 75% of the text segments in the corpus for this analysis, since those without active words or without the minimum size are disregarded^(13)^. Of the total number of text segments in the corpus (356), 76.97% were considered for this analysis, with an average of 32.12 occurrences per text segment. In fact, each of the classes has a main idea, which was further developed by presenting the typical text segments.

## RESULTS

The responses of the care-seekers were organized into a single corpus. Firstly, the characterization of the care-seekers is presented, followed by the descending hierarchical classification (DHC) and the text segments of each class.

### Characterization of the researched-caregivers

Of the total of 24 caregivers, the majority were male (87.50%), single (54.17%), illiterate (41.67%), retired (79.17%), with an income of one minimum wage (62.50%), Catholic (75.00%), and predominantly older adult (62.50%). Detailed information on the caregivers participants can be seen in Table 1.

**Table 1:** Distribution of the characteristics of the caregivers participants

Variables	f	%
Gender		
Male	21	87.50
Female	3	12.50
Marital status		
Single	13	54.17
Married/stable union	5	20.83
Divorced	3	12.50
Widowed	3	12.50
Schooling		
Illiterate	10	41.67
Literate	3	12.50
1st year Primary school	3	12.50
2nd year Primary school	2	8.33
3rd year Primary school	1	4.17
4th grade Elementary school	2	8.33
8th grade Elementary school	2	8.33
High school	1	4.17
Occupation		
Retired	19	79.17
Beneficiary	2	8.33
Pensioner	2	8.33
Unemployed	1	4.17
Income		
None	1	4.17
1 salary	15	62.50
2 salaries	4	16.67
3 salaries	4	16.67
Religion		
Catholic	18	75.00
Spiritist	1	4.17
Evangelical	3	12.50
No religion	2	8.33

Chart 1 details the presentation of the thematic axes, with their respective classes and needs apprehended from the researched-caregivers to facilitate understanding and organization of the corpus.

**Chart 1:** Presentation of the thematic axes, classes and perceived needs of the researched-caregivers

AXES	CLASSES	PERCEIVED NEEDS
1. Description of transitional processes.	1. Developmental and health-disease transitions	Process, perception and ruptures.
	5. Situational and organizational transitions	Process of communicating the diagnosis.
2. Understanding coping strategies for transitional processes.	2. Conditions that facilitate and inhibit transitions.	Resignation of caregivers in relation to sequelae.
	4. Transition process indicators.	Acceptance of health condition with quality of life assessment.
	3. Transition result indicators.	Experiencing transitions and the need to adapt.

**Figure 1:**
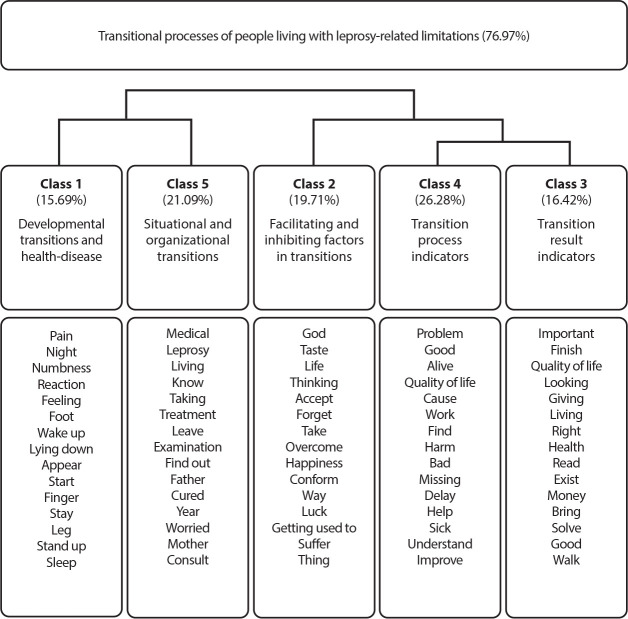
Dendrogram of transitional process classes

Initially, the corpus goes through a series of consecutive divisions, which form the classes. At first, the corpus is separated into two blocks of text segments, the first block is divided into Classes 1 and 5, while the second is divided into Class 2 and a new block of text segments, which are finally divided into Classes 4 and 3, as can be seen in the Figure 1.

### Axis 1: Description of transitional processes

This axis describes the transitional processes experienced by the researched-caregivers, and is made up of classes 1 and 5.

### Class 1: Developmental and health-disease transitions - process, perception and ruptures

The first class to be highlighted in the analysis was class 1. This is made up of 43 text segments (15.69%), and from the associated words it is possible to infer that aspects related to pain and the first symptoms of leprosy are addressed. The segments were:

*His fingers and ears began to thicken and his legs became tired. Anything that hit hurt. I couldn’t sleep at night. And so it went, slowly.*

*It’s because the numbness started in my fingers. It went from the fingers to the hands. And it went away. Then there was numbness in my legs and feet. I couldn’t work anymore. I couldn’t do any more work. I had to be admitted to the Hospital Colônia and I’m still here today.*

*The sequelae of leprosy have appeared in both my feet, they’re numb. It never went away. It’s a permanent sequel, and to this day I haven’t found a remedy to make it better. No way. That’s how I feel. It’s bad to live like this.*

### Class 5: Situational and organizational transitions - diagnostic communication process

Class 5 has 60 text segments (21.90%), and the words associated with it mention the initial diagnosis of leprosy. As for the text segments in Class 5, we can see that:

*The next day I had my tests and they showed leprosy. I asked the doctor what leprosy was and he told me that leprosy was the ancient leprosy before Christ. I wasn’t surprised. I was amazed at all the patients here.*

*I thought I was going to die, that there was no cure, but I asked the doctor and the social worker a lot of questions, I even asked if I was going to be cured and they told me that if I did the six-month treatment, I would be cured.*

[...] *and discovered that I had leprosy. It was a fright for me, I was a child, and I was abandoned inside the Colony Hospital, with no family, no one, nothing. It was very difficult to get used to, but I got used to it.*

### Axis 2: Understanding coping strategies for transitional processes

This axis shows the patterns of responses with the apprehension of coping strategies for transitional processes. These patterns are divided into facilitating and inhibiting conditioning factors, process and outcome indicators of transitional processes. Classes 2, 4 and 3 were presented in this axis.

### Class 2: Facilitating and inhibiting factors of transitions - resignation of the researched-caregivers in relation to the sequelae

Class 2 is made up of 54 text segments (19.71%), and the associated words refer to the resignation of the researched-caregivers in relation to the sequelae of leprosy, as well as highlighting the role of spirituality in this process. In this context, distancing may have been the way in which the researched-caregivers dealt with the diagnosis and treatment, as can be seen in:

*One year of treatment, one year, one month and fifteen days of treatment inside the Colony Hospital, in isolation, which was the isolation at the time, called a leprosarium. And in ways that have changed my life to this day.*

*I have to lead my life the way God wants me to, because that’s how you have to lead your life: accept it, be patient, calm, that everything will come right.*

*The disease hit me very hard, but I lead my life with these sequelae, I suffer prejudice, people don’t talk to us properly, they don’t look us in the eye, but I have God, I pray every day at dawn, and so I go on living, with the strength of my God and loneliness makes me sad, it’s the sadness of needing something and not having someone around. As you know, God created man and then woman, because he saw that Adam needed a woman, and he said that Eve would be a helper.*

### Class 4: Process indicators of transitions - acceptance of health condition with evaluation of quality of life

Class 4 groups together 72 text segments (26.28%), and the words associated with it mention the processes related to leprosy that affect quality of life. It looked at the acceptance of the respondents’ current health condition - care with quality of life after the diagnosis of leprosy. It can be seen in segments of text that the research participants indicate that they have a good quality of life:

*I think my quality of life is good. I’m alive, my family is at peace, I receive visits from my daughters, so I can say that I have a satisfactory quality of life, thank God.*

*I think my quality of life is good, despite all the problems and difficulties. The really bad thing is not being able to work anymore, a person who has been used to working since he was young and finds himself in a situation without a salary, without being able to work is bad and I can think that my quality of life is good, since I’m alive, it’s just a bit complicated not being able to have a fixed income, a job, or a pension, that’s too bad, it harms a person’s quality of life.*

### Class 3: Transition outcome indicators - experiencing transitions and the need to adapt

In Class 3 there are 45 text segments (16.42%) and the associated words refer to what the researched-caregivers consider to be important for quality of life, taking into account the experience of transitions and the need to adapt to the new health situation, as observed in the text segments:

*Having quality of life is very important. It’s so important that every day I ask God to give me a healthy life, with more health, because when I got leprosy, it came on very strong, I thought I wasn’t going to live, I cried a lot, thinking I was going to die.*

*It’s important for us to have a good quality of life, but with the sequelae of leprosy, we don’t get to have it, so nothing happens.*

*Quality of life is having one more day of life every day, it’s receiving one more day from God, one more night in your life, that’s too important, there are days when I think about eliminating myself, but I go back and see that it’s not worth it.*

## DISCUSSION

During the data analysis process, it was possible to identify the transitions experienced by the researched-caregivers, in the light of Meleis’ Transition Theory^(8)^. In this way, the researched-caregivers experienced the four types of transitions, including feelings of fear, worry, loneliness, hopelessness, guilt and a tendency to hide the diagnosis of leprosy because they were unaware of how it was transmitted.

Class 1 identified the developmental and health-disease transitions, with the process, perception and ruptures being the perceived needs. Process, perception and rupture are universal characteristics of transition. The process refers to the duration of the transition, i.e. the period between the anticipation of the need to change and stability in the new condition. Perception refers to the relationship with meaning for those experiencing a transition, influencing the results of the transition. Finally, disruption refers to the break with people’s relationships, commitments, references and expectations ^(14)^.

A study carried out in the northeast of Brazil, in order to understand the repercussions of leprosy on the lives of children and adolescents who have experienced the process of becoming ill, showed repercussions on daily life, related to the diagnosis, the fear of being discovered, the perspective of having the identity of being healthy re-signified, the experience of prejudice and the construction of a negative social life, with repercussions on social relationships^(15)^.

This thought is in line with the study carried out in southern Brazil to understand the repercussions of leprosy on the daily lives of people living with the disease, where changes were observed in daily life, changes in routine, in the family environment, in the performance of work activities and in lifestyle. Consequently, after diagnosis, people experienced feelings of fear, sadness and hopelessness, resulting in physical, social and emotional changes^(16)^, reinforcing the experience of the universal characteristics of transitions.

In class 5, the situational and organizational transitions were identified as the process of communicating the diagnosis, leaving home as a form of treatment, the first reactions and concerns about the diagnosis and the family. Situational transition occurs when a person is inserted into a certain environment as a result of the changes that have taken place and requires a redefinition of roles. Organizational transition includes institutional changes and can be precipitated by changes in the political, social, economic and personal spheres^(17)^.

In this way, the study showed social isolation with institutionalization, which was the treatment in force. Certainly, in Brazil, the policy of compulsory isolation practiced with people affected by leprosy between the 1920s and 1980s segregated those affected by the disease. Therefore, the trajectory experienced by these people within the colony institutions was marked by physical, institutional, social and psychological changes. Even leaving the institution was equally traumatic, due to their unpreparedness for life outside it^(18)^. In view of this, it is pointed out that the researched-caregivers experienced the situational and organizational transitions, since they were compulsorily hospitalized, remaining in the institution to this day, and even though they experienced the policy of compulsory isolation, they developed healthy relationships with each other, overcame disabilities and cultivated dreams and desires, aware of their role in society.

Class 2, on the other hand, emphasizes the facilitating and inhibiting factors of the transitions, and highlights the resignation of the research participants-caregivers to the consequences of leprosy, which led to physical and mental limitations.

The way of dealing with a transition differs from person to person, and is influenced by various factors in the transitional course, which can facilitate or inhibit a given transition.

It should also be noted that the physical disability caused by leprosy is preventable and reversible if detected early, and can occur due to the nerve damage caused by the inflammation triggered by the presence of Mycobacterium leprae. Disabilities are chronic and can worsen throughout life, even after treatment has been completed. Disability leads to deformities, thus perpetuating low self-esteem, exclusion, discrimination and social stigma^(19)^.

A review study carried out to analyze the evidence of how social stigma compromises the mental health of people with leprosy found that social stigma acts as a psychosocial stress factor, affecting emotional health and causing psychological damage to well-being and self-esteem. Mental distress, anxious and depressive symptoms, suicidal thoughts, intense suffering and social isolation were found, which can be aggravated in the presence of sequelae, affecting the ability to work and activities of daily living^(20)^.

In this way, it is emphasized that the sequelae had a negative personal meaning for the researched-caregivers, acting as inhibiting conditions for healthy transitions, in addition to the stigma associated with the experience of having leprosy. Resignation, faith and hope were also highlighted as cultural beliefs and attitudes among the research participants in the face of change. These beliefs and attitudes were shown to facilitate transitional processes.

Class 4 shows the process indicators of transitions, emphasizing the acceptance of the health condition and the assessment of quality of life. According to Meleis et al.^(7)^, process indicators include: feeling involved, interacting, being situated, developing confidence and coping.

It was observed that the researched-caregivers showed involvement and interaction with family and friends, comparison of their previous life with their current one, including acceptance of their health condition, confidence to deal with their new condition and the ability to make decisions in relation to their new way of living. Expectations changed, but in a conscious way, with the acquisition of knowledge, a sense of adaptation and acceptance, with a consequent evaluation of quality of life.

In this context, a scoping study aimed at exploring the scientific evidence related to the quality of life of people with leprosy showed that the greatest impairment of quality of life was related to the delay in diagnosing the disease, leprosy reactions, physical disabilities, neuropathic pain and stigma^(9)^.

Acceptance of emerging difficulties also refers to acceptance of the state of health and requires the construction of meanings, new meanings and perceptions attributed to the transitional process experienced.

Class 3 revealed the outcome indicators of the transitions, showing the experience of transitions and the need to adapt. The outcome indicators are twofold: the mastery of new skills and the reformulation of identities, and represent the end of a transition. The mastery of new competences develops with the incorporation of skills and behaviours along the transitional path to manage the new situation^(7)^.

It was revealed that the researched-caregivers demonstrated mastery of new skills and reformulation of identities, and consequently experienced the transitional processes in a healthy way, since they acquired new skills, restructured daily routines, attributed meanings to the transitional process, new priorities and adapted to the new situation by integrating the changes into their way of life.

In addition, it should be mentioned that transition is a period that can generate imbalance, stress or confusion, however, when a new beginning is reached, in the end, a new situation ensues^(21)^, more fluid and coherent with the reality experienced. For this to happen, we need a professional eye that takes time and is attentive to all situations of change.

In fact, the changes that took place with the caregivers affected their quality of life and required them to adapt to their new condition, generating instability in the personal, work, spiritual, social and psycho-emotional spheres. As a result, transitional care was essential and had a major impact on the lives of the research participants, since it enabled emotional support, the development of positive coping strategies, improvements in health and well-being, encouragement for family involvement and an individualized care plan.

Therefore, in this study it is possible to infer that the transitions were experienced in a healthy way, since the text segments presented made it possible to ascertain the adaptation to the new life, with the consequent achievement of quality of life.

### Study limitations

A limitation of the study is that it was carried out during the post-transition period, and it was not possible for the researcher-caregiver to carry out therapies to anticipate the critical events that cause transitions. In this way, we worked on promotion, prevention and intervention to develop effective transition paths, based on an understanding of the experiences lived.

### Contributions to Nursing, Health or Public Policy

It provides support for the professional practice of nurses and other health professionals, by giving greater visibility to the transitions experienced, ruptures, situations of rejection and discrimination that can affect physical and mental health.

## FINAL CONSIDERATIONS

The transitional processes had a positive meaning, as they helped them adapt to and accept a new way of living, incorporating and experiencing a new routine and new roles. The researched-caregivers experienced developmental, health-disease, situational and organizational transitions, with multiple, sequential and related patterns, with awareness of physical, social, family, environmental and economic changes.

The results of this study point to the need for future research that considers aspects related to the empowerment and protagonism of people with limitations resulting from leprosy, as well as with other chronic diseases that affect emotional, physical and social well-being.
