# Supplementary material for: Transitions experienced by people living with limitations resulting from leprosy: a research-care study
Source: Rev Bras Enferm. 2024 Nov 22;77(5):e20230229. doi: 10.1590/0034-7167-2023-0229 (PMC11653868; doi:10.1590/0034-7167-2023-0229)
Supplement: Translation

# Translation: Transições vivenciadas por pessoas que convivem com limitações decorrentes da hanseníase: uma pesquisa-cuidado

**DOI:** 10.1590/0034-7167-2023-0229pt

**Authors:** Rayla Maria Pontes Guimarães Costa, Marcia Astrês Fernandes, Ivete Palmira Sanson Zagonel

**Affiliations:** I Universidade Estadual do Piauí. Parnaíba, Piauí, Brasil; II Universidade Federal do Piauí. Teresina, Piauí, Brasil; III Universidade Federal do Paraná. Curitiba, Paraná, Brasil

**Keywords:** Hanseníase, Pessoas com Deficiência, Adaptação Psicológica, Cuidado Transicional, Qualidade de Vida

## Abstract

**Objetivo:**

compreender os processos transicionais que incidem na adaptação de pessoas que convivem com limitações decorrentes da hanseníase.

**Métodos:**

pesquisa de abordagem qualitativa fundamentada nos preceitos da Teoria de Transição, mediada pela pesquisa-cuidado, com 24 pessoas com limitações decorrentes da hanseníase em um ex-hospital colônia, no Piauí. Foram realizadas entrevistas semiestruturadas. Procedeu-se a análise com suporte do *software Iramuteq.*

**Resultados:**

os pesquisados-cuidados vivenciaram os quatro tipos de transições, inclusive, experimentaram sentimentos de medo, preocupação, solidão, desesperança, culpa e tendência a esconder o diagnóstico. Desvelou-se rupturas e resignação, tendo a espiritualidade, a adaptação à nova situação de vida e aceitação, como condições facilitadoras do enfrentamento do processo transicional, com consequente melhoria da qualidade de vida.

**Considerações Finais:**

os processos transicionais tiveram significado positivo, visto que contribuíram na adaptação e no alcance da qualidade de vida.

## INTRODUÇÃO

A hanseníase é uma doença infectocontagiosa, causada pelo bacilo *Mycobacterium leprae*, que ataca pele, mucosas e nervos periféricos, e tem potencial de ocasionar lesões neurais, com danos irreversíveis. Dessa forma, essas lesões neurais desencadeadas pela hanseníase, são responsáveis pelas incapacidades, levando ao estigma e discriminação. A hanseníase representa um importante problema de saúde publica no Brasil, que ocupa, atualmente, a segunda posição no mundo entre os países que registram casos novos^(1)^.

Além disso, a hanseníase ainda é transmitida ativamente em vários países, com destaque para a Índia, o Brasil e a Indonésia, que são responsáveis por cerca de 74,5% do total de casos registrados em 2021 (140.594). Cada um desses países notificou mais de 10.000 casos^(2,3)^, o que requer uma atenção especial, uma vez que a doença pode evoluir com limitações.

As limitações decorrentes da hanseníase afetam o cotidiano do paciente, pois impactam na realização das atividades de vida diária, necessitando de atenção dos profissionais de saúde antes mesmo do início do tratamento^(4)^. De fato, é imperioso desenvolver a prática profissional da enfermagem com pessoas que convivem com limitações provenientes da hanseníase, ocupando-se da escuta e do cuidado profissional, considerando as subjetividades, compreendendo as transições vivenciadas diante de mudanças que exigem o enfrentamento e a adaptação à nova situação de vida.

Em relação ao processo de adaptação, ele pode ocorrer de maneira tardia ou repentinamente em articulação com as mudanças que ocorrem ao longo do ciclo vital, ocasionadas por intercorrências vivenciais^(5)^, que podem afetar a qualidade de vida da pessoa em processo de transição.

A transição pode ser conceituada como a passagem de uma fase, lugar, condição/estado e/ou assunto para outro, em decorrência de uma mudança. Enquanto processo e resultado, ela surge da interação entre pessoa e ambiente, encontra-se agregada em um contexto e/ou situação, expressando uma mudança no estado de saúde ou nas relações de papéis, expectativas ou habilidades^(6)^, estando associada a outros conceitos como adaptação, ajustamento e autocuidado^(7)^, em que as respostas a esse processo são manifestadas por comportamentos relacionados à saúde^(8)^.

Assim sendo, foi possível conjecturar uma lacuna na assistência integral voltada para pessoas com limitações em virtude da hanseníase na perspectiva das transições ocorridas no processo de adoecimento, que comprometeram o bem-estar e a qualidade de vida, afetando, sobremaneira, o modo de viver dessas pessoas, podendo causar vulnerabilidade e marcas na autoestima, nos aspectos cognitivos e neuropsiquiátricos.

Salienta-se, ainda, a relevância científica e social do estudo, sobretudo considerando que, no aspecto científico, a pesquisa é inovadora e contribui significativamente para o avanço no que tange ao desenvolvimento de estratégias no trato com pacientes portadores da hanseníase. Socialmente, aborda-se o desdobramento de uma doença antiga que, até os dias atuais, continua causando impacto negativo nas pessoas afetadas. Inclusive, seus efeitos incidem diretamente na qualidade de vida, podendo causar danos, tanto físicos quanto psicológicos. No aspecto físico, interfere na realização das tarefas diárias e laborais, enquanto no aspecto psicológico afeta as relações sociais no ambiente em que se vive, podendo levar à exclusão, solidão e até mesmo à depressão^(9)^.

Dessa maneira, buscou-se focalizar as transições vivenciadas por essas pessoas ao longo de suas vidas. Nesse contexto, definiu-se como objeto de estudo as transições vivenciadas por sujeitos que convivem com limitações decorrentes da hanseníase e a adaptação às mudanças, na perspectiva da pesquisa-cuidado. Pressupõe-se, que o objeto subsidiará a construção de novos conhecimentos e questionamentos inerentes às transições vivenciadas. Além disso, o estudo proposto visa responder à seguinte questão norteadora: qual a significação dos processos transicionais que incidem na adaptação de pessoas que convivem com limitações decorrentes da hanseníase pela pesquisa-cuidado?

## OBJETIVO

Compreender os processos transicionais que incidem na adaptação de pessoas que convivem com limitações decorrentes da hanseníase.

## MÉTODOS

### Aspectos éticos

O estudo foi conduzido de acordo com as diretrizes de ética nacionais e internacionais e aprovado pelo Comitê de Ética em Pesquisa da Universidade Federal do Piauí (UFPI). Cumpriu-se os preceitos éticos na execução e desenvolvimento da pesquisa, cujo parecer está anexado à presente submissão.

### Tipo de estudo e referencial teórico-metodológico

Pesquisa de abordagem qualitativa ancorada nos preceitos da Teoria de Transição, uma teoria de enfermagem, de médio alcance, baseada nos processos transicionais experimentados ao longo do ciclo de vida do ser humano^(8)^, mediada pelos aspectos metodológicos da pesquisa-cuidado. A pesquisa-cuidado pode se desenvolver em cinco etapas: aproximação com o objeto de estudo; encontro da pesquisadora (cuidadora) com o ser pesquisado (cuidado); estabelecimento das conexões da pesquisa, teoria e prática do cuidado; afastamento dos seres pesquisador e pesquisado e análise do apreendido^(10)^. Utilizou-se todas as etapas da pesquisa-cuidado no presente estudo.

Ressalta-se que para a pesquisa-cuidado é essencial que o enfermeiro (ser pesquisador) assegure uma relação de cuidado com o ser pesquisado/cuidado, onde, pesquisa e cuidado fazem parte de um mesmo contexto, com isso, o enfermeiro, em um momento, ouve e registra e, em outro, cuida e não registra, cuida e registra, educa, gerencia, ou seja, pesquisa enquanto cuida-ensina-gerencia^(11)^. Além disso, foram seguidas as recomendações para a divulgação de estudos qualitativos, proposto pelo guia *Consolidated criteria for reporting qualitative research* (COREQ)^(12)^.

### Cenário do estudo

O estudo foi realizado em um ex-hospital colônia, nosocômio público estadual, referência no tratamento da hanseníase no estado do Piauí, localizado no município de Parnaíba, Piauí.

### Fonte de dados

Os participantes do estudo foram 24 pessoas com idade a partir de 18 anos, que apresentam limitações em decorrência da hanseníase. Os critérios de inclusão foram: apresentar sequelas permanentes com diferentes graus de limitação e ter disponibilidade e interesse em participar do estudo. Foram excluídos do estudo os que estiveram ausentes do local do estudo durante o período da coleta de dados.

### Coleta e organização dos dados

A coleta de dados ocorreu no período de junho a setembro de 2021, utilizando-se a entrevista semiestruturada de forma presencial. Dessa forma, foi utilizado como instrumento, para guiar a entrevista, um roteiro com os seguintes tópicos: significado do diagnóstico da hanseníase, convivência com as limitações advindas do contágio com a doença, alterações/mudanças observadas na vida a partir do diagnóstico, avaliação da qualidade de vida após o diagnóstico da doença, problemas vivenciados em decorrência das limitações que afetaram a qualidade de vida e estratégias utilizadas para superação dos problemas/mudanças. Ainda, os dados também incluíram o perfil sociodemográfico de cada participante (idade, sexo, estado civil, escolaridade, ocupação, renda e religião).

A entrevista ocorreu por meio de encontros realizados na instituição, de forma individualizada. Nesse encontro, explicou-se a temática e o objetivo da pesquisa, bem como, a técnica de coleta de informações que seriam adotadas. Realizou-se o convite para participação na pesquisa, após o consentimento, agendou-se a entrevista em data e horário adequados ao ser pesquisado-cuidado, e solicitou-se a anuência para participação no estudo. Assim, o Termo de Consentimento Livre e Esclarecido (TCLE) foi obtido de todos os pesquisados-cuidados por meio escrito. As entrevistas foram realizadas em local reservado, conforme agendamento. Concluída a entrevista, procedeu-se à prática individualizada do cuidado transicional. As entrevistas foram gravadas em aparelho MP4 e transcritas na íntegra.

Inclusive, nesse momento, ocorreu a aplicação da pesquisa-cuidado propriamente dita, em que se deu a interação entre ser pesquisador e o ser pesquisado. Desse modo, o pesquisador-cuidador se ocupou em captar e desvelar as significações atribuídas pelas pessoas que convivem com limitações decorrentes da hanseníase perante as transições vivenciadas e adaptação na perspectiva do alcance da qualidade de vida. Concretizou-se o cuidado de variadas formas: escuta, olhar, proximidade, respeito, inclusão, orientações de saúde e acompanhamento. Nesse momento, houve expressão de emoções, como tristeza e alegria, ao rememorar aspectos da própria vida, ou seja, as vivências e reações a determinadas situações.

Destarte, os dados foram organizados em dois eixos temáticos e apresentados em cinco classes, com as respectivas necessidades apreendidas durante a concretização da pesquisa-cuidado.

### Análise dos dados

As informações oriundas das entrevistas foram gravadas e transcritas na íntegra. Posteriormente, foram analisadas com o auxílio do *software Iramuteq* (*Interface de R pourles Analyses Multidimensionnelles de Textes et de Questionnaires*, versão 0.7), um programa gratuito e de fonte aberta criado por *Pierre Ratinaud* que permite a explicação gráfica de dados qualitativos^(13)^.

Inicialmente, as entrevistas foram transcritas e organizadas em um único *corpus* textual. Para isso, algumas palavras foram agrupadas, no intuito de melhorar a análise. Outras palavras, como, por exemplo, “qualidade de vida”, “problemas vivenciados” e “mudanças” foram mantidas. Durante a organização do material para análise das variáveis sociodemográficas como idade, gênero, estado civil, escolaridade, ocupação, renda e religião. Tais aspectos foram incluídos na linha de comando e apresentados em uma tabela.

Para a análise, utilizou-se a Classificação Hierárquica Descendente (CHD) e os dados foram apresentados em uma figura. Outrossim, tendo em vista a robustez da Classificação Hierárquica Descendente (CHD), autores indicam uma retenção mínima de 75% dos segmentos de texto do *corpus* para essa análise, visto que aqueles sem palavras ativas ou sem o tamanho mínimo são desconsiderados^(13)^. Do total de segmentos de texto do *corpus* (356), 76,97% foram considerados para essa análise, tendo, em média, 32,12 ocorrências por segmentos de texto. Inclusive, cada uma das classes apresenta uma ideia principal, a qual foi aprofundada por meio da apresentação dos segmentos de textos típicos.

## RESULTADOS

As respostas dos pesquisados-cuidados foram organizadas em um único *corpus*. Primeiramente, apresenta-se a caracterização dos pesquisados-cuidados, em seguida, a classificação hierárquica descendente (CHD) e os segmentos de texto de cada classe.

### Caracterização dos pesquisados-cuidados

Do total de 24 pesquisados-cuidados, a maioria é do sexo masculino (87,50%), são pessoas solteiras (54,17%), analfabetas (41,67%), aposentadas (79,17%), com renda de um salário mínimo (62,50%), católicas (75,00%), e, predominantemente, idosos (62,50%). As informações detalhadas sobre os pesquisados-cuidados podem ser verificadas na Tabela 1.

**Tabela 1:** Distribuição das características dos pesquisados-cuidados

Variável	f	%
Sexo		
Masculino	21	87,50
Feminino	3	12,50
Estado Civil		
Solteiro(a)	13	54,17
Casado/União estável	5	20,83
Divorciado	3	12,50
Viúvo	3	12,50
Escolaridade		
Analfabeto(a)	10	41,67
Alfabetizado(a)	3	12,50
1º ano Ensino fundamental	3	12,50
2º ano Ensino fundamental	2	8,33
3 º ano Ensino fundamental	1	4,17
4 º ano Ensino fundamental	2	8,33
8 º ano Ensino fundamental	2	8,33
Ensino médio	1	4,17
Ocupação		
Aposentado(a)	19	79,17
Beneficiário	2	8,33
Pensionista	2	8,33
Desempregado	1	4,17
Renda		
Nenhuma	1	4,17
1 salário	15	62,50
2 salários	4	16,67
3 salários	4	16,67
Religião		
Católica	18	75,00
Espírita	1	4,17
Evangélico	3	12,50
Sem religião	2	8,33

Detalha-se, no Quadro 1, a apresentação dos eixos temáticos, com suas respectivas classes e necessidades apreendidas dos pesquisados-cuidados para facilitar o entendimento e organização do corpus.

**Quadro 1:** Apresentação dos eixos temáticos, classes e necessidades apreendidas dos pesquisados-cuidados

EIXOS	CLASSES	NECESSIDADES APREENDIDAS
1. Descrição dos processos transicionais.	1.Transições desenvolvimental e de saúde-doença.	Processo, percepção e rupturas.
	5. Transições situacional e organizacional	Processo de comunicação do diagnóstico.
2. Apreensão das estratégias de enfrentamento dos processos transicionais.	2. Condicionantes facilitadoras e inibidores das transições.	Resignação dos pesquisados-cuidados em relação às sequelas.
	4. Indicadores de processo das transições.	Aceitação da condição de saúde com avaliação da qualidade de vida.
	3. Indicadores de resultado das transições.	Vivência das transições e necessidade de adaptação.

Inicialmente, o *corpus* passa por uma série de divisões consecutivas, as quais formam as classes. A princípio, o *corpus* se separa em dois blocos de segmentos de texto, o primeiro bloco se divide nas Classes 1 e 5, ao passo que o segundo se divide na Classe 2 e em um novo bloco de segmentos de texto, que, por fim, se dividem nas Classes 4 e 3, como pode ser observado na Figura 1.

**Figura 1:** Dendrograma de classes dos processos transicionais

### Eixo 1: Descrição dos processos transicionais

Este eixo descreve os processos transicionais vivenciados pelos pesquisados-cuidados, e é formado pelas classes 1 e 5.

### Classe 1: Transições desenvolvimental e de saúde-doença - processo, percepção e rupturas

A primeira classe a ser destacada na análise foi a classe 1. Esta é formada por 43 segmentos de texto (15,69%) e, a partir das palavras associadas, é possível inferir que são abordados aspectos relacionados a dores e aos primeiros sintomas da hanseníase. Os segmentos foram:

*Os dedos e as orelhas começaram a ficar grossos, e as pernas, cansadas. Qualquer coisa que batia, doía. Faltava o sono à noite. E foi assim, devagarzinho.*

*É porque começou uma dormência nos dedos. Dos dedos, passou para as mãos. E passou. E ficou a dormência nas pernas e nos pés. Não pude mais trabalhar. Não pude mais fazer serviço nenhum. Tive que vir me internar aqui no Hospital Colônia e estou aqui até hoje.*

*A sequela da hanseníase apareceu nos meus dois pés, são dormentes. Nunca passou. É uma sequela definitiva, até hoje não encontrei remédio para ficar bom. De jeito nenhum. É isso aí que eu sinto. É ruim viver assim.*

### Classe 5: Transições situacional e organizacional - processo de comunicação do diagnóstico

Por sua vez, a Classe 5 agrupa 60 segmentos de texto (21,90%), e as palavras associadas a ela fazem menção ao diagnóstico inicial da hanseníase. Quanto aos segmentos de texto da Classe 5, é possível verificar:

*No dia seguinte, fiz os exames e acusou hanseníase, perguntei ao médico o que era hanseníase e ele me disse que hanseníase era a antiga lepra antes de Cristo. Não fiquei espantado. Eu fiquei espantado com todos os pacientes que tinham aqui.*

*Achava que ia morrer, que não tinha cura, mas fiz muitas perguntas ao médico e à assistente social, cheguei a perguntar se eu ia ficar curado e me disseram que se eu fizesse o tratamento de seis meses, ficaria curado.*

[...] *e descobriu que eu estava com hanseníase. Para mim foi um susto, eu era uma criança, e fiquei abandonado dentro do Hospital Colônia, sem família, sem ninguém, sem nada. Foi muito difícil me acostumar, mas me acostumei.*

### Eixo 2: Apreensão das estratégias de enfrentamento dos processos transicionais

Este eixo mostra os padrões de respostas com a apreensão das estratégias de enfrentamento dos processos transicionais. Esses padrões dividem-se em condicionantes facilitadores e inibidores, indicadores de processo e de resultado dos processos transicionais. Neste eixo, foram apresentadas as classes 2, 4 e 3.

### Classe 2: Condicionantes facilitadores e inibidores das transições - resignação dos pesquisados-cuidados em relação às sequelas

A Classe 2 é formada por 54 segmentos de texto (19,71%), e as palavras associadas fazem referência à resignação dos pesquisados-cuidados em relação às sequelas da hanseníase, bem como destaca o papel da espiritualidade no processo. Nesse contexto, o distanciamento pode ter sido a forma como os pesquisados-cuidados lidaram com o diagnóstico e com o tratamento, como pode ser visto em:

*Um ano de tratamento, um ano, um mês e quinze dias de tratamento dentro do Hospital Colônia, isolado, que era o isolamento na época, chamado leprosário. E de maneiras que a minha vida, até hoje, ela mudou por um todo.*

*Eu tenho que levar como Deus quer, porque assim que você tem que levar a sua vida: aceitar, ter paciência, calma, que tudo vai vencer.*

*A doença me baqueou muito, mas levo minha vida com essas sequelas, sofro preconceitos, as pessoas não conversam com a gente direito, não olham nos olhos, mas tenho Deus, oro todos os dias de madrugada e, assim, vou vivendo, com a força de meu Deus e solidão me deixa triste, é a tristeza de você precisar de alguma coisa e não tem uma pessoa por perto. Como você sabe Deus criou o homem e depois a mulher, porque viu que Adão precisava de uma mulher, e Ele disse que Eva ia ser uma auxiliadora, ajudadora.*

### Classe 4: Indicadores de processo das transições - aceitação da condição de saúde com avaliação da qualidade de vida

A Classe 4 agrupa 72 segmentos de texto (26,28%), e as palavras associadas a ela fazem menção aos processos relacionados à hanseníase que afetam a qualidade de vida. Averiguou-se a aceitação da condição de saúde atual dos pesquisados-cuidados com a qualidade de vida após o diagnóstico da hanseníase. Pode-se observar em segmentos de texto que os pesquisados-cuidados indicam possuir uma boa qualidade de vida:

*Eu acho boa a minha qualidade de vida. Estou viva, minha família está em paz, recebo visitas das minhas filhas, então, posso afirmar que tenho qualidade de vida satisfatória, graças a Deus.*

*Acho que minha qualidade de vida é boa, apesar de todos os problemas e dificuldades. O ruim mesmo é a gente não poder trabalhar mais, uma pessoa que era acostumada a trabalhar desde novo e se achar numa situação sem ordenado, sem poder trabalhar, é ruim, e consigo pensar que minha qualidade de vida é boa, já que estou vivo, só é um pouco complicado não poder ter uma renda fixa, um trabalho, ou um aposento, isso é ruim demais, prejudica a qualidade de vida da pessoa.*

### Classe 3: Indicadores de resultado das transições - vivência das transições e necessidade de adaptação

Na Classe 3 há 45 segmentos de texto (16,42%) e as palavras associadas fazem menção ao que os pesquisados-cuidados julgam ser importantes para a qualidade de vida, levando em consideração o vivenciar das transições e a necessidade de adaptação à nova situação de saúde, como observado nos segmentos de texto:

*Ter qualidade de vida é muito importante. Tão importante que todos os dias peço a Deus para me dar uma vida saudável, com mais saúde, pois quando peguei hanseníase, ela veio muito forte, pensei que não fosse viver, chorava muito, achando que eu ia morrer.*

*É importante a gente ter uma boa qualidade de vida, mas com as sequelas da hanseníase a gente não chega a ter, possuir, então, nada feito.*

*Qualidade de vida é você ter um dia a mais de vida todos os dias, é você receber de Deus um dia a mais, uma noite a mais na sua vida, isso é importante demais, tem dias que penso em me eliminar, mas volto atrás e vejo que não vale a pena.*

## DISCUSSÃO

Durante o processo de análise dos dados foi possível identificar as transições vivenciadas pelos pesquisados-cuidados, à luz da Teoria de Transição de Meleis^(8)^. Dessa maneira, os pesquisados-cuidados vivenciaram os quatro tipos de transições, inclusive, experimentaram sentimentos de medo, preocupação, solidão, desesperança, culpa e tendência a esconder o diagnóstico da hanseníase por desconhecimento da forma de contágio.

Na classe 1, identificou-se as transições desenvolvimental e de saúde-doença, sendo o processo, a percepção e as rupturas as necessidades apreendidas. O processo, a percepção e a ruptura são características universais da transição. O processo alude-se à duração da transição, ou seja, ao período entre a antecipação da necessidade de mudar e a estabilidade na nova condição. A percepção refere-se à relação com o significado para quem experiencia uma transição, influenciando os resultados da transição. Por fim, a ruptura relaciona-se ao rompimento com as relações, compromissos, referências e expectativas das pessoas^(14)^.

Um estudo realizado no nordeste do Brasil, a fim de compreender as repercussões da hanseníase na vida de crianças e adolescentes que vivenciaram o processo de adoecimento, mostrou repercussões no cotidiano, relacionadas ao diagnóstico, pelo receio de ser descoberto, na perspectiva de ter a identidade de sadio ressignificada, além de uma vivência de preconceito e construção de vida social negativa, com repercussão nas relações sociais^(15)^.

Este pensamento coaduna com o estudo realizado no sul do Brasil, com o intuito de compreender as repercussões da hanseníase no cotidiano das pessoas que vivem com a doença, em que se observou mudanças no cotidiano, alterações na rotina, no ambiente familiar, no desempenho das atividades laborais e no estilo de vida. Consequentemente, após o diagnóstico, as pessoas experimentaram sentimentos de medo, tristeza, e desesperança, resultando em alterações físicas, sociais e emocionais^(16)^, reforçando a vivência das características universais das transições.

Na classe 5, ao apresentar as transições situacional e organizacional, identificou-se o processo de comunicação do diagnóstico, onde se deu a saída de casa como forma de tratamento, as primeiras reações e as preocupações com o diagnóstico e a família. A transição situacional ocorre quando uma pessoa é inserida em determinado meio em decorrência das mudanças ocorridas e requer a redefinição de papéis. A transição organizacional compreende mudanças institucionais e podem ser precipitadas por mudanças no âmbito político, social, econômico e pessoal^(17)^.

Dessa maneira, no estudo em tela, mostrou-se o isolamento social com a institucionalização, que era o tratamento em vigor. Certamente, no Brasil, a política de isolamento compulsório praticada com as pessoas acometidas pela hanseníase, entre as décadas de 1920 e 1980, segregou os acometidos pela doença. Logo, a trajetória vivenciada por essas pessoas, dentro das instituições colônias, foi marcada por mudança física, institucional, social e psicológica. Inclusive, o desligamento da instituição era igualmente traumático, em virtude do despreparo para a vida fora dela^(18)^. Em vista disso, pontua-se que os pesquisados-cuidados vivenciaram as transições situacional e organizacional, uma vez que foram internados compulsoriamente, permanecendo, até os dias atuais na instituição, e mesmo tendo vivenciado a política de isolamento compulsório, desenvolveram relações saudáveis entre si, superaram as deficiências e cultivaram sonhos e desejos, conscientes de seu papel na sociedade.

Já na classe 2, enfatiza-se os condicionantes facilitadores e inibidores das transições, apreendeu-se a resignação dos pesquisados-cuidados com as sequelas da hanseníase, que acarretaram as limitações físicas e mentais.

A maneira de lidar com uma transição difere de pessoa para pessoa, e é influenciada por diversos fatores no decurso transicional, que podem facilitar ou inibir uma dada transição.

Salienta-se ainda que a incapacidade física gerada pela hanseníase é evitável e reversível se detectada precocemente, e pode ocorrer devido à lesão nervosa gerada pela inflamação desencadeada pela presença do *Mycobacterium leprae*. As deficiências são crônicas e podem se agravar ao longo da vida, mesmo após a conclusão do tratamento. A deficiência leva a deformidades, perpetuando, assim, a baixa autoestima, a exclusão, a discriminação e o estigma social^(19)^.

Um estudo de revisão realizado com o intuito de analisar as evidências de como o estigma social compromete a saúde mental das pessoas com hanseníase, constatou-se que o estigma social atua como um fator de estresse psicossocial, afetando a saúde emocional e causando danos psíquicos ao bem-estar e à autoestima. Foram encontrados sofrimento mental, sintomas ansiosos e depressivos, pensamentos suicidas, sofrimento intenso e isolamento social, que podem ser agravados na presença de sequelas, afetando a capacidade para o trabalho e para as atividades de vida diária^(20)^.

Desse modo, enfatiza-se que as sequelas tiveram um significado pessoal negativo nos pesquisados-cuidados, atuando como condições inibidoras das transições saudáveis, além do estigma associado à experiência de ter hanseníase. Pontua-se, ainda, a resignação, a fé e a esperança como crenças e atitudes culturais nos pesquisados-cuidados frente às mudanças. Tais crenças e atitudes revelaram-se facilitadoras dos processos transicionais.

Na classe 4, aponta-se os indicadores de processo das transições, enfatizando a aceitação da condição de saúde e a avaliação da qualidade de vida. Para Meleis et al.^(7)^, os indicadores de processo incluem: sentir-se envolvido, interagir, estar situado, desenvolver confiança e enfrentamento.

Observou-se que os pesquisados-cuidados manifestaram envolvimento e interação com familiares e amigos, comparação da vida anterior com a atual, inclusive com aceitação da condição de saúde, confiança para lidar com a nova condição e capacidade para tomada de decisões em relação ao novo modo de viver. As expectativas mudaram, mas de forma consciente, com aquisição de conhecimentos, senso de adaptação e de aceitação, com consequente avaliação da qualidade de vida.

Nesse contexto, um estudo de revisão de escopo, cujo objetivo era explorar as evidências científicas relacionadas à qualidade de vida de pessoas com hanseníase, evidenciou que o maior comprometimento da qualidade de vida se relacionou ao atraso no diagnóstico da doença, às reações hansênicas, às incapacidades físicas, à dor neuropática e ao estigma^(9)^.

Outrossim, a aceitação das dificuldades emergentes refere-se à aceitação do estado de saúde e à demanda para a construção de significados, novos sentidos e percepções atribuídas ao processo transicional vivenciado.

Na classe 3, revelou-se os indicadores de resultado das transições, constatando o vivenciar das transições e a necessidade de adaptação. Os indicadores de resultado são dois: o domínio de novas competências e a reformulação de identidades, representando o fim de uma transição. O domínio de novas competências desenvolve-se com a incorporação de habilidades e condutas ao longo do percurso transicional para gerir a nova situação^(7)^.

Revelou-se que os pesquisados-cuidados demostraram o domínio de novas competências e reformulação de identidades, consequentemente, vivenciaram os processos transicionais de maneira saudável, visto que houve a aquisição de novas habilidades, reestruturação de rotinas diárias, atribuição de significados ao processo transicional, novas prioridades e adaptação à nova situação com integração das mudanças no modo de viver.

Além disso, é necessário mencionar que a transição é um período que pode gerar desequilíbrio, estresse ou confusão, entretanto, quando um novo começo é alcançado, no final, sucede-se uma nova situação^(21)^, mais fluida e coerente com a realidade vivida. Para que isso ocorra, é preciso um olhar profissional demorado e atento a todas as situações de mudanças.

De fato, as mudanças ocorridas com os pesquisados-cuidados afetaram a qualidade de vida e exigiu adaptação à nova condição e gerou instabilidade nos âmbitos pessoal, laboral, espiritual, social e psicoemocional. Com isso, o cuidado transicional foi primordial e impactou, sobremaneira, na vida dos pesquisados-cuidados, uma vez que possibilitou apoio emocional, desenvolvimento de estratégias de enfrentamento positivas, melhorias na saúde e no bem-estar, incentivo ao envolvimento familiar e plano de cuidado individualizado.

Portanto, neste estudo é possível inferir que as transições foram vivenciadas de maneira saudável, uma vez que os segmentos de texto apresentados permitiram averiguar a adaptação à nova vida, com consequente alcance da qualidade de vida.

### Limitações do Estudo

Como limitação do estudo, menciona-se a realização durante o período de pós-transição, não sendo possível ao pesquisador-cuidador realizar terapêuticas de antecipação aos eventos críticos causadores das transições. Dessa forma, trabalhou-se a promoção, a prevenção e a intervenção para desenvolver caminhos de transições eficazes, a partir da compreensão das experiências vivenciadas.

### Contribuições para a Área da Enfermagem, Saúde ou Política Pública

Oferece subsídio para a prática profissional de enfermagem e dos demais profissionais de saúde, ao possibilitar maior visibilidade das transições vivenciadas, rupturas, situações de rejeição, discriminação que podem afetar a saúde física e mental.

## CONSIDERAÇÕES FINAIS

Os processos transicionais tiveram significado positivo, uma vez que auxiliaram na adaptação e aceitação da nova forma de viver, com incorporação e vivência de nova rotina e novos papéis. Os pesquisados-cuidados vivenciaram transições desenvolvimental, saúde-doença, situacional e organizacional, com padrões múltiplos, sequenciais e relacionados, com consciência das mudanças físicas, sociais, familiares, ambientais e econômicas.

Os resultados deste estudo apontam para a necessidade de futuras pesquisas que considerem aspectos referentes à capacitação e ao protagonismo de pessoas com limitações decorrentes da hanseníase, bem como, com outras doenças crônicas, que afetam o bem-estar emocional, físico e social.
